# Oesophageal pneumatosis in a case of bowel ischaemia

**DOI:** 10.1259/bjrcr.20170039

**Published:** 2017-07-29

**Authors:** Milena Pasquali, Stefano Leonardo, Miria Morsiani, Maurizio Zompatori

**Affiliations:** ^1^Department of Experimental, Diagnostic and Specialty Medicine (DIMES), Division of Radiology, S.Orsola-Malpighi Hospital, Bologna, Italy; ^2^Department of Radiology, Santa Maria della Scaletta Hospital, Imola, Bologna, Italy; ^3^Department of Radiology, Cardiothoracic section, Policlinico Sant Orsola-Malpighi, Bologna, Italy

## Abstract

Pneumatosis intestinalis (PI) is a condition in which cystic collections of gas develop within the gastrointestinal wall, forming submucosal or subserosal “bubbles”. The radiologic manifestations are often dramatic and most notably are associated with life-threatening bowel ischaemia. PI may occur as a primary type but is usually secondary in nature, attributable to a wide spectrum of causes (benign and fulminant), ranging from immunosuppression to bowel infarction. Herein, we report a case of massive and extensive PI in a patient with small bowel ischaemia, having both benign and serious clinical origins.

## Clinical presentation

An 80-year-old female arrived at our emergency department, presenting with diffuse, unrelenting abdominal pain, nausea and vomiting. According to relatives, the pain had begun the prior evening, intensifying during the following day. Her current medical problems included chronic obstructive bronchopulmonary disease (COPD), treated by oxygen therapy and steroids; atrial fibrillation (AF), treated by anticoagulants; and mild kidney failure. All vital signs were viewed acceptable by staff physicians, but the abdomen was tense and very painful. Other than white blood cell count (15,000 mm^–3^), C-reactive protein (18 mg dl^–1^) and serum creatinine (2.5 mg dl^–1^) levels, laboratory data were non-contributory.

## Investigations/imaging findings

**Figure 1.**
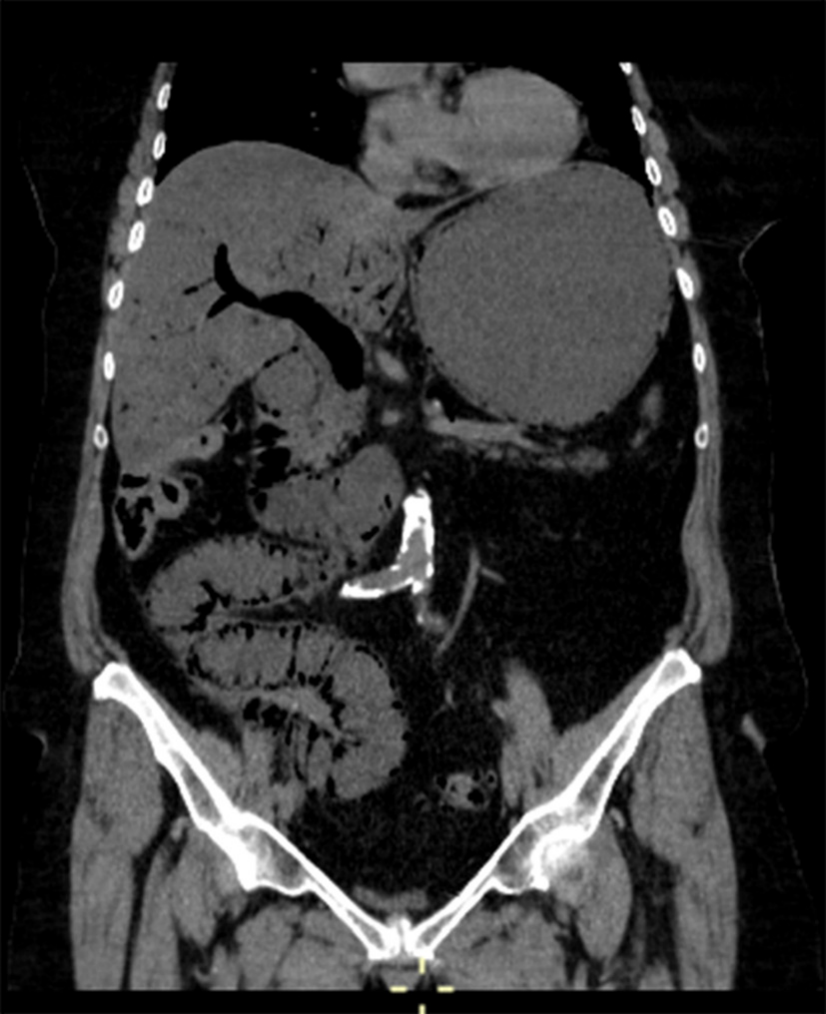
CT scan (coronal multiplanar reformatted, abdominal window): Massive pneumatosis of small bowel and gastric walls, voluminous portomesenteric venous gas and mild fat stranding between loops of bowel.

**Figure 2.**
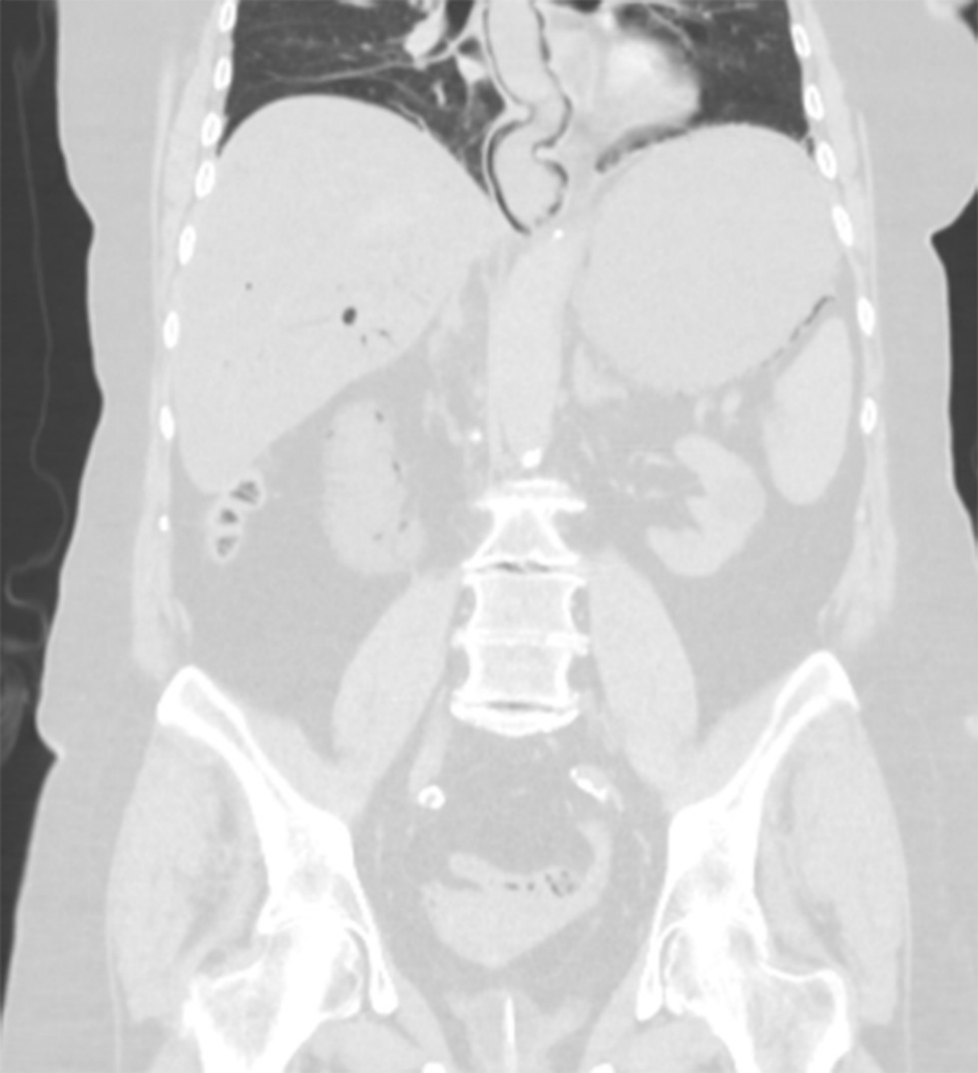
CT scan (coronal multiplanar reformatted, lung window): Copious air dissecting the entire wall of the oesophagus (partly included in scan).

**Figure 3.**
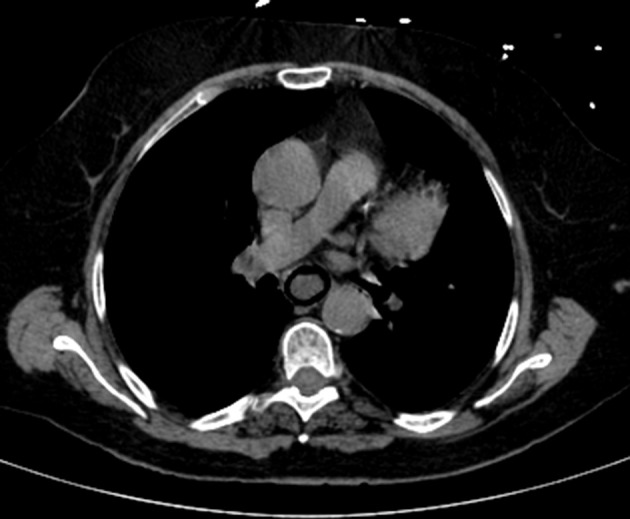
CT scan (axial multiplanar reformatted, abdominal window): Circumferential pneumatosis of the oesophageal wall.

A CT abdominal scan was then performed, foregoing use of contrast medium. The CT images were highly characteristic of PI, which was massive in this instance. There was intramural gas involving most of the small bowel and stomach, and a considerable amount of portomesenteric endoluminal gas had reached the intrahepatic branches and splenic vein (Figure 1). A minimal amount of free peritoneal fluid and mesenteric stranding were reported as well. The wall of the oesophagus also was overtaken by gas, a rare finding in PI (Figure 2). Just partly visible in CT images, the oesophagus was completely encircled by gas in axial views, creating an “air-donut” effect (lumen at the centre) (Figure 3).

**Figure 4.**
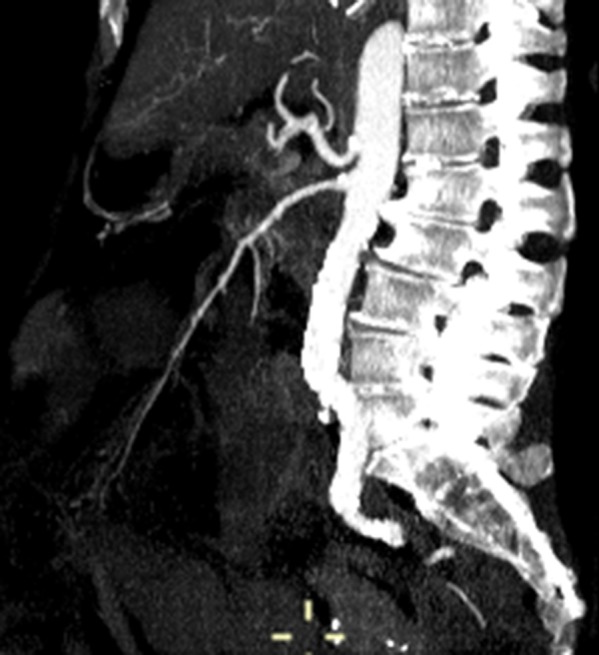
CT scan (sagittal multiplanar reformatted, maximum intensity projection reconstruction): All major abdominal vessels appear patent and well opacified by contrast material.

After adequate hydration, the patient underwent a second CT scan the next morning, this time with injection of contrast medium. All major splanchnic vessels were shown to be patent (Figure 4).

## Treatment

Nevertheless, surgical intervention was abandoned, given the radiographic evidence of advanced intestinal necrosis and the multiple comorbidities cited by relatives. Supportive measures only were instituted.

## Outcome

A few hours after the final CT images were obtained, the patient died.

## Discussion

The most common cause of PI and portomesenteric air is bowel ischaemia (70% of cases), which carries a high mortality rate of up to 75–90%.^1^

PI is commonly described as a finding of transmural bowel ischaemia.

If portomesenteric gas is also present, the specificity for ischeamic bowel approaches 100%. Thus, despite the many non-emergent scenarios associated with PI, those causes with life-threatening potential must first be excluded.^2^

The aetiology and pathogenesis of PI are not fully understood but are likely multifactorial.^3–4^

PI probably occurs owing to a disruption of mucosal integrity, and there are two major theories about the source of the intramural gas.^5^

The first involves submucosal translocation of gas-producing luminal bacteria through mucosal defects, either gaps or hyperpermeable segments (bacterial theory). The second premise is that non-inflamed bowel wall is dissected by the normally present luminal gas, owing to lost mucosal integrity (mechanical theory).

The course of an ischaemic event is triphasic. During the initial stage (0–6 h), the most common finding is intense, acute abdominal pain, associated often with diarrhoea and shock. Symptoms then typically subside, marking a silent phase (7–12 h) where dull abdominal pain, intestinal paralysis, and rapidly deteriorating status prevail. During the final phase (12–24 h), ileus and bacterial peritonitis with sepsis are evident, and multiorgan failure ensues.^6^

Both theories of PI development are feasible under these circumstances.

In our patient, the most relevant finding was the massive amount of gas involving not only the small bowel but also the stomach and oesophagus. Axial CT images of oesophagus even showed circumferential intramural gas, imparting an air-donut effect. Otherwise, CT findings suggested intestinal ischaemia as the predisposing condition, based on acknowledged clinical infirmities and despite confirmed patency of all intestinal vessels. One may therefore assume that vaso-occlusive intestinal ischaemia was the fundamental issue, with no residual thrombus in a patient taking anticoagulants for atrial fibrillation. In the absence of generalized hypoperfusion, non-occlusive vascular ischaemia is less tenable.

On the other hand, if transmural ischaemic necrosis is key in the development of PI, the implication that a fleeting vascular occlusion accounts for such extensive gastrointestinal involvement must be questioned. There are, however, ancillary explanations for the voluminous intramural air detected in the stomach and oesophagus of this patient. Had superior mesenteric arterial occlusion taken place, leading to intestinal necrosis and ileus, then compromised bowel, distended and taut from stagnant secretions and burgeoning bacterial gas, would be the likely impetus for broad propagation of PI upstream to the oesophageal level.^7^

Another equally plausible supposition is that multiple factors are at play. It may be that transmural intestinal necrosis (due to vascular ischaemia) and adverse tissue effects of long-term corticosteriod exposure (for COPD) acted in concert to produce widespread PI.^7–9^

Prolonged steroid therapy is known to deplete intestinal lymphoid populations, thus impairing gastrointestinal defences, reducing peristalsis and compromising the integrity of the intestinal wall.^9,10^

## Conclusions

Pneumatosis intestinalis is an entity with multiple aetiologies and may be associated with a fatal outcome. When associated with the presence of portomesenteric venous gas, it is typically the result of bowel ischaemia. Abdominal radiography and CT scanning are the most frequently used techniques for the diagnosis of PI. But CT is the best imaging modality for establishing the diagnosis of PI, as denoted by findings such as intramural gas parallel to the bowel wall.^11^

Therefore, it is essential that radiologists identify worrisome clinical situations, alerting emergency personnel and collaborating with patient management teams.^12^

Some authors have suggested that cystic or bubbly PI is apt to be innocuous, whereas linear gas collections have more severe implications. However, it is not necessarily true that radiologic appearances are predictive of disease severity.^13^ Although mesenteric stranding, intestinal wall thickening and distension, ascites and confinement of intramural gas to small bowel are considered troublesome CT features of PI, often neither these traits nor documented portomesenteric venous gas are definitive signs of transmural bowel infarction after ischaemic insults. The clinical outcomes in such scenarios seem to hinge largely on the gravity and extent of any underlying disease.^14^

## Learning points

1. In this patient with extensive PI, oesophageal pneumatosis was observed as a particularly rare event. This finding was demonstrable in axial CT images, presenting an appearance of “air donut.”
2. A combination of pneumatosis, portomesenteric venous gas and predisposing health issues is particularly ominous.
3. If pneumatosis is massive, both life-threatening events (*i.e*. bowel ischaemia) and lesser predisposing causes (corticosteroids, COPD) may be contributory.

## Consent

Written informed consent was obtained from the relatives (sons) of our patient for publication of this case report, including accompanying images.
